# Eutrophication strengthens the response of zooplankton to temperature changes in a high‐altitude lake

**DOI:** 10.1002/ece3.2308

**Published:** 2016-08-30

**Authors:** Yun Li, Ping Xie, Dandan Zhao, Tianshun Zhu, Longgen Guo, Jing Zhang

**Affiliations:** ^1^ Fisheries CollegeHuazhong Agricultural UniversityWuhan430070HubeiChina; ^2^ Donghu Experimental Station of Lake EcosystemsState Key Laboratory of Freshwater Ecology and BiotechnologyInstitute of HydrobiologyChinese Academy of SciencesWuhan430072HubeiChina

**Keywords:** Cyanobacterial blooms, detrended correspondence analysis, eutrophication, path analysis, temperature, zooplankton

## Abstract

To assess whether and how zooplankton communities respond to variations in temperature and how these assemblages change with eutrophication, we performed a large‐scale, monthly survey from August 2011 to July 2012 to determine the seasonal and spatial variations in these communities in a high‐altitude lake. A detrended correspondence analysis and a path analysis demonstrated that temperature and chlorophyll a were important factors influencing zooplankton. The path diagram showed that *Daphnia* was negatively affected directly by chlorophyll a and indirectly by temperature, whereas *Bosmina* was directly and positively affected by temperature. *Daphnia* spp. decreased in both absolute and relative biomass during warm seasons, whereas *Bosmina* spp. showed the opposite trend. Moreover, the lowest *Daphnia* spp. biomass was observed in the southern region, which was the most eutrophic. Our results indicate that increasing temperatures will continue to shift the dominant genus from *Daphnia* to *Bosmina*, and this change will be exacerbated by eutrophication. In addition, the zooplankton of Lake Erhai have shifted to smaller species over time as temperature and eutrophication have increased, which implies that zooplankton succession to small cladocerans may be markedly accelerated under further climate change and the increased eutrophication that has been observed in recent decades.

## Introduction

Warming can increase the susceptibility of biotic communities to trophic cascade effects, eutrophication, and the combined impacts of other anthropogenic perturbations (Sala et al. 2000; Kratina et al. 2012). In freshwater ecosystems, warming can induce variations in the species composition, body size, and abundance of plankton communities (Moore and Folt 1993; Gardner et al. 2011; Meerhoff et al. 2012; Domis et al. 2013). The effects of seasonal fluctuations in temperature on zooplankton should be studied because temperature influences these communities by promoting species growth rates and reproductive success during warm seasons (Van der Have and De Jong 1996; Kingsolver and Huey 2008).

High temperatures influence zooplankton by not only reducing their size at maturity but also shortening their reproductive cycles (Hanazato and Masayuki 1885). Additionally, temperatures higher than those required for optimal growth have been shown to negatively affect zooplankton, such as *Daphnia* spp. (Moore et al. 1996; Strecker et al. 2004), by reducing their ingestion efficiency (Rall et al. 2010). In contrast, Messner et al. (2013) found that elevated temperatures can enhance zooplankton biomass and species diversity in some species by increasing growth rates (Hall and Burns 2002), which might lead to greater grazing pressure on phytoplankton (O'Connor et al. 2009). Moreover, temperature fluctuations might influence bottom‐up effects by altering the physiological features and population structure of phytoplankton communities by, for example, increasing the growth rate of toxic cyanobacteria in the summer (Johnk et al. 2008). Abundant toxic cyanobacteria in warm seasons would change the composition of the zooplankton community (Hall et al. 1976).

The influence of temperature on the zooplankton community changes with eutrophication (Kratina et al. 2012). In freshwater, eutrophication results in high concentrations of chlorophyll a (Chl a) and abundant phytoplankton (Ptacnik et al. 2008; Jochimsen et al. 2012; Huo et al. 2013). In addition, elevated temperatures promote the growth of *Microcystis* because it has a higher optimal temperature compared with most other phytoplankton species (Coles and Jones 2000; Johnk et al. 2008) and a higher tolerance to low transparency environments caused by buoyant cyanobacteria (Huisman et al. 2004), which are typically abundant in eutrophic waters. Abundant phytoplankton benefit zooplankton by providing high food availability and inhibit large cladocerans because harmful and nutritionally poor species account for a large proportion of the phytoplankton biomass (Tillmanns et al. 2008; Domis et al. 2014; Filstrup et al. 2014). Therefore, eutrophication should not be neglected when discussing the effects of temperature variation on zooplankton, and additional, thorough investigations involving large‐scale field surveys in natural systems are required to determine how zooplankton communities change under eutrophic conditions.

Recently, high‐altitude lakes have been widely studied for their ecological sensitivity to environmental fluctuations (Manca and Armiraglio 2002), such as climate change (Nevalainen and Luoto 2012; Angeler et al. 2013), human activities (Garcon et al. 2012), and exotic species invasions (Moreira et al. 2015). Therefore, temperature variations and eutrophication might cause more dramatic changes in the zooplankton communities of high‐altitude lakes. The seasonal succession of zooplankton communities is thought to be triggered primarily by temperature and light (Sommer et al. 2012). In this study, a high‐altitude lake was selected for investigation because the high levels of solar radiation it receives year‐round (Laurion et al. 2000) should minimize the effects of light supply on the zooplankton communities.

In this study, we conducted a large‐scale survey of Lake Erhai, a high‐altitude lake, to explore whether and how zooplankton communities respond to variations in temperature and how these responses are influenced by eutrophication. We investigated the seasonal and spatial variability in the zooplankton community of the lake and then analyzed the changes in the structure and dynamics of this community in response to temperature and the combined effects of other environmental perturbations, such as Chl a.

## Materials and Methods

### Study area

Lake Erhai (100°05′‐100°17′E, 25°36′‐25°58′N) is located in the central zone of Dali Bai Autonomous Prefecture, Yunnan Province, China, and is the second largest high‐altitude freshwater lake on the Yunnan Plateau. The lake has a surface area of approximately 251 km^2^, an elevation of 1974 m, and a volume of nearly 28.8 × 10^8^ m^3^, and the average and maximum water depths are 10.5 and 20.9 m, respectively. Since the 1950s, the lake has been affected by anthropogenic eutrophication caused by the increased population density surrounding the lake, which is highest around the southern region of the lake and gradually decreases toward the northern region.

Cyanobacterial blooms first appeared in 1957 in the southern region of Lake Erhai (Li et al. 1963), and large‐scale cyanobacterial blooms appeared in 1996 and 1998 with *Anabaena* spp. dominating the cyanobacteria population. Cyanobacteria blooms then spread throughout the entire lake except for the northern bay (Dong 1999), but the dominant cyanobacteria species during the warm seasons shifted to *Microcystis* spp. after 2008 (Wen and Ma 2011).

### Sampling and analysis

**Figure 1:**
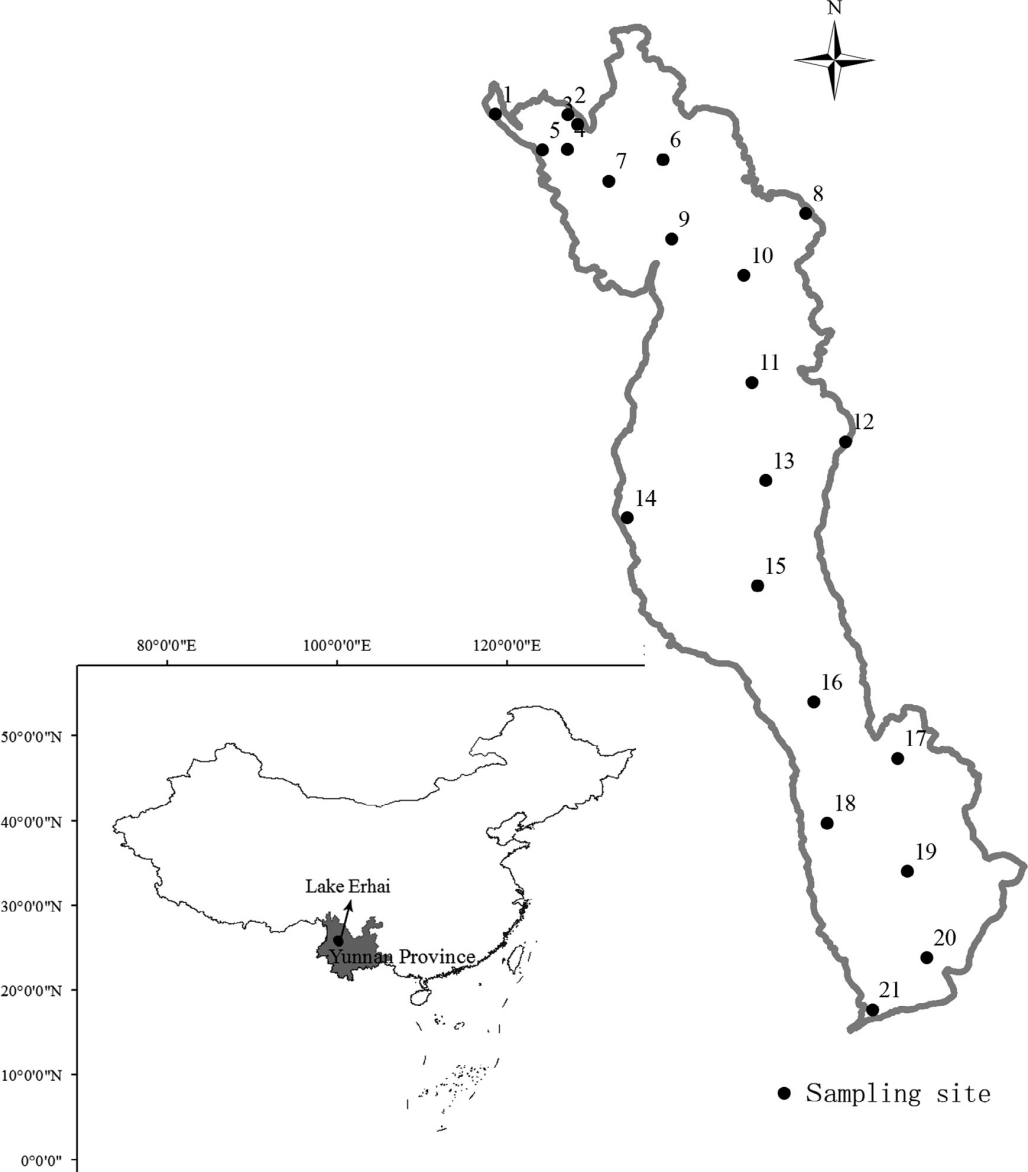
Lake Erhai sampling sites.

Sampling in Lake Erhai was conducted monthly from August 2011 to July 2012 at 21 stations distributed throughout the entire lake. The stations were randomly distributed within the northern regions (stations 1–9), the central regions (stations 10–15), and the southern regions (stations 16–21) (Fig. 1).

Water samples were collected from each site at the upper (i.e., 0.5 m below the water surface), middle (midway between the surface and the bottom), and lower (i.e., 0.5 m above the sediment surface) parts of the water column and then pooled together for subsequent analyses of the hydrochemical parameters and plankton communities. Total nitrogen (TN), total phosphorus (TP), nitrate nitrogen (NO_3_‐N), ammonia nitrogen (NH_4_‐N), phosphate phosphorus (PO_4_‐P), and Chl a of each sample were measured in the laboratory according to the methods detailed by Huang et al. (1999), and 1‐L water samples were preserved in acetic Lugol's solution and concentrated to 50 mL after sedimentation for 48 h in Utermohl chambers to analyze the phytoplankton and rotifers (Huang et al. 1999). The phytoplankton in 0.1‐mL samples were counted and measured under 400× magnification using an Olympus microscope (Olympus, Tokyo, Japan), and an ultrasonic crusher (JY88‐II; Scientiz, Ningbo, Zhejiang, China) was used to separate and count the single cells of the *Microcystis* colonies. Taxonomic identification of the phytoplankton was performed according to Hu (2006). The rotifers in 1‐mL samples were counted and measured under 200× magnification using an Olympus microscope and identified according to Voigt and Koste (1978). The crustaceans in 10‐L water samples were sieved through 64‐*μ*m plankton nets and preserved with 5% formalin for further analysis (Huang et al. 1999). In these samples, all of the individuals were counted and identified according to Shen et al. (1979) and Chiang and Du (1979), and where possible, the bodies of at least 30 individuals of each species were measured under 40× magnification with an Olympus microscope. The biomass of each plankton species was calculated using the methods described by Huang et al. (1999).

The water temperature (T), pH value, dissolved oxygen (DO), and conductivity (COND) were measured onsite at 0.5 m below the water surface with a YSI ProPlus multiparameter water quality meter (Yellow Springs, OH). The Secchi depth (SD) was assessed with a black and white Secchi disk (20 cm in diameter) to determine water transparency.

### Statistical analyses

Multiple competing hypotheses were applied to assess whether specific environmental variables affected the succession of zooplankton. First, a detrended correspondence analysis (DCA) was performed using CANOCO 5.0 (Braak and Šmilauer 2002) to assess the effects of the environmental variables on the zooplankton community composition (relative biomasses) with a short gradient length. The assessed environmental variables were T, TN, TP, N:P (TN/TP ratio), NO_3_‐N, NH_4_‐N, PO_4_‐P, DO, SD, pH, Chl a, total phytoplankton biomass, and *Microcystis* biomass. The dependent variables were the relative biomasses of rotifers, cladocerans, copepods, *Daphnia hyalina*,* Bosmina longirostris*, and *Ceriodaphnia quadrangula*. After forward selection, only the significant independent variables (*P *<* *0.05) were included in the final DCA ordination, which showed that the explanatory variables accounted for 32.6% of the variation in the zooplankton community data. Second, path analysis was conducted to test the specific effects of the environmental variables on zooplankton succession using AMOS software version 21.0 (SPSS, Inc., Chicago, IL). The variables in the final model were only selected if they had significant causal relationships with the main zooplankton categories. A chi‐square test was performed to assess the alignment between the original correlation matrix and the overall model, in which a high *P*‐value (>0.05) indicates that the data fit the model well. Third, a linear regression analysis was employed to gain a greater understanding of the linkage between the relative biomass of the dominant zooplankton categories and the most important environmental variables. The linear regression was implemented in R using the car package, and a leverage plot was produced (Sall 1990). To isolate the effects of specific environmental factors on zooplankton, we first removed the effects of time and space using generalized linear models (GLMs). We used time, longitude and latitude as covariates, and each zooplankton category as a dependent variable in the GLMs with maximum likelihood estimation, and the resulting residual variation was then used as the target variable in the DCA, path analysis, and linear regression analysis (Legendre 1993; Ziegler et al. 2015). A one‐way ANOVA was conducted to test whether there were significant differences in the abiotic parameters among the different regions in SPSS version 19.0 for Windows software (SPSS, Inc.). All of the data were tested for homogeneity and normality, where these assumptions were violated; the data were log10‐transformed prior to performing the statistical analysis.

## Results

### Seasonal and spatial variations in the zooplankton community

**Figure 2:**
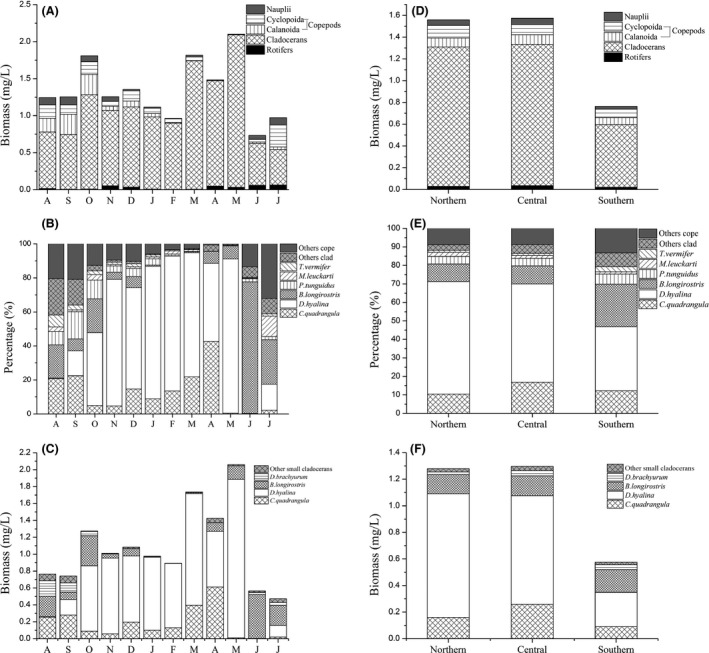
Seasonal and spatial variation in the zooplankton community. Seasonal variation: (A) zooplankton biomass, (B) biomass of the main species as a fraction of the total crustacean zooplankton biomass, and (C) cladoceran biomass. Spatial variation: (D) zooplankton biomass, (E) biomass of the main species as a fraction of the total crustacean zooplankton biomass, and (F) cladoceran biomass. To determine seasonal variation, the average biomass for station numbers 1 to 21 was calculated for each month. To determine spatial variation, the average biomass over 12 months was calculated for each site.

In this study, we identified a total of 30 rotifer taxa and 23 crustacean zooplankton taxa. Cladocerans dominated the zooplankton community from August 2011 to July 2012, and the dominant species included *Daphnia hyalina* (*D. hyalina*), *B. longirostris*, and *C. quadrangula*. The main copepod species were *P. tunguidus* and *T. vermifer*; rotifers accounted for only a small proportion of the community in this study (Fig. 2A,D).

The biomass of cladocerans was low from August to September 2011 and June to July 2012, which was mainly because of the reduction in *D. hyalina* (Fig. 2A–C), which was the most abundant species in most seasons (except August to September 2011 and June to July 2012). *C. quadrangula* was also abundant in certain months, and *B. longirostris* and other cladocerans increased from August to September 2011 and June to July 2012, with *B. longirostris* constituting 77.4% of the total crustacean biomass in June 2012 (Fig. 2B). In addition, the absolute and relative biomass values of copepods were comparatively high from August to October 2011 and in July 2012 (Fig. 2A,B). Regarding the spatial variations, the absolute and relative biomass of the cladocerans and *Daphnia* spp. decreased in the southern region (Fig. 2A–C), but the relative biomass of copepods and *Bosmina* spp. increased (Fig. 2E).

### Seasonal and spatial variations in abiotic parameters and Chl a

**Figure 3:**
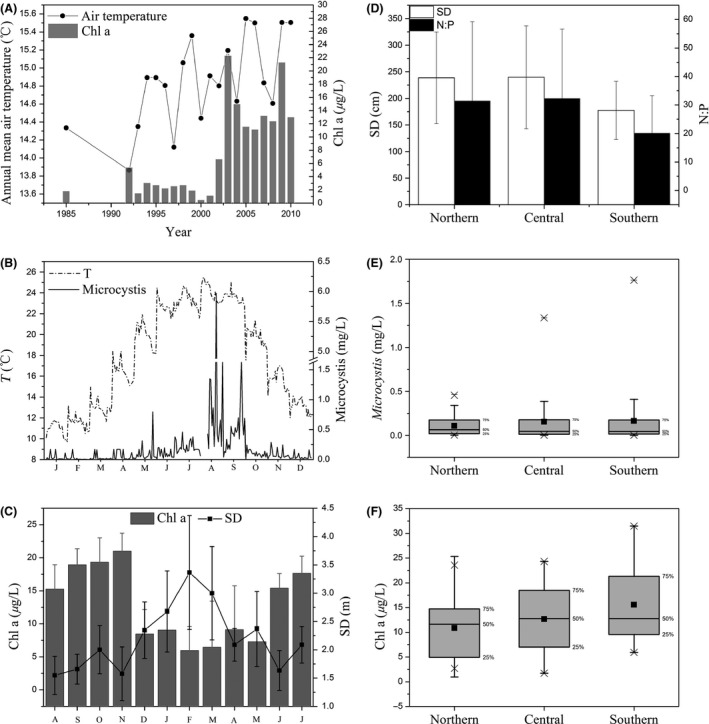
Temporal and spatial variation in abiotic parameters, Chl a, and *Microcystis* in Lake Erhai. (A) Long‐term changes in the air temperature and Chl a from 1985 to 2010; (B) *Microcystis* biomass and water temperature (T) from August 2011 to July 2012 and monthly data for all sites from the northern region to the southern region; (C) Chl a and SD from August 2011 to July 2012; (D) SD and N:P; (E) *Microcystis* biomass; (F) Chl a. Data for A were obtained from the literature (Huang et al. (2013), Zhao et al. (2011) and Du et al. (1987).

Clear seasonal variations in temperature were observed, with the highest values (>20°C) occurring from August to September 2011 and from June to July 2012 (Fig. 3B). The seasonal variations in Chl a and SD exhibited opposite trends, with the highest Chl a (mean value of 18.0 *μ*g·L^−1^) and the lowest SD values found from August to November 2011 and from June to July 2012 (Fig. 3C).

The means and ranges of the abiotic parameter and Chl a values in different regions during the study are presented in Table 2. Significant spatial variations in water temperature were not observed (*P *=* *0.315, Table 1). Compared with the other parameters, Chl a exhibited significantly higher spatial variability (*P *<* *0.001, Table 1), and the mean Chl a value over all sampling sites was 12.8 *μ*g·L^−1^. The highest Chl a value was observed in the southern region and the lowest in the northern region (Fig. 3F). SD and N:P were lowest in the southern region and were not significantly different between the northern and central regions (*P *>* *0.05, Fig. 3D).

**Table 1:** Results of one‐way ANOVAs of the environmental parameters and *Microcystis* in three gradients from August 2011 to July 2012, with time as a covariate

	T		Chl a		SD		N:P		*Microcystis*	
	*F*	*P*	*F*	*P*	*F*	*P*	*F*	*P*	*F*	*P*
Region	1.162	0.315	15.894	**<0.001**	12.568	**<0.001**	6.002	**0.003**	0.992	0.374

T, water temperature; Chl a, chlorophyll a concentration; SD, Secchi depth; N:P, total nitrogen to total phosphorous ratio.

Significant effects are indicated in bold.

All of the data were log (*x* + 1)‐transformed prior to analysis to meet normality and homogeneity of variance assumptions.

### Seasonal and spatial variations in *Microcystis*

The peak in *Microcystis* biomass, 5.97 mg·L^−1^, occurred from August to September 2011, but the biomass was low in the other months (Fig. 3B). The mean *Microcystis* biomass from August 2011 to July 2012 for all of the sampling sites was 0.14 mg·L^−1^. Spatially, the mean *Microcystis* biomass was not significantly different among the different regions (*P *=* *0.374, Table 1), but the minimum‐to‐maximum ranges clearly varied, with the widest range 0.004 to 1.76 mg·L^−1^, occurring in the southern region (Fig. 3E, Table 2).

**Table 2:** Mean values and ranges of abiotic parameters, Chl a and *Microcystis* in the different regions of Lake Erhai from August 2011 to July 2012

	Northern, mean (range)	Central, mean (range)	Southern, mean (range)	All, mean (range)
*Microcystis* (mg·L^−1^)	0.11 (0.002–0.55)	0.15 (0.002–1.33)	0.16 (0.0004–1.76)	0.14 (0.0004–1.76)
Chl a (*μ*g·L^−1^)	10.5 (1.0–25.3)	12.7 (1.7–24.3)	15.9 (5.9–31.5)	12.8 (1.0–31.5)
TN (mg·L^−1^)	0.68 (0.04–1.59)	0.67 (0.15–1.65)	0.65 (0.06–1.42)	0.67 (0.04–1.65)
TP (mg·L^−1^)	0.031 (0–0.108)	0.041 (0–0.485)	0.040 (0–0.218)	0.037 (0–0.485)
PO_4_‐P (mg·L^−1^)	0.012 (0–0.052)	0.012 (0–0.026)	0.010 (0–0.048)	0.011 (0–0.052)
NO_3_‐N (mg·L^−1^)	0.19 (0–1.08)	0.14 (0.04–0.57)	0.15 (0.06–0.56)	0.16 (0–1.08)
NH_4_‐N (mg·L^−1^)	0.08 (0–0.48)	0.08 (0–0.45)	0.08 (0.01–0.47)	0.08 (0–0.48)
SD (cm)	239 (50–480)	240 (110–500)	177 (100–330)	221 (50–500)
T (°C)	17.9 (10.1–25.2)	18.0 (10.9–24.1)	17.1 (9.6–23.6)	17.7 (9.6–25.2)
DO (mg·L^−1^)	7.5 (4.6–10.3)	7.5 (5.3–8.8)	7.7 (5.9–9.4)	7.6 (4.6–10.3)
COND (*μ*s·cm^−1^)	263.6 (24.16–563.0)	252.2 (211.7–308.6)	242.2 (183.5–309.8)	254.5 (24.2–563.0)
pH	8.7 (8.1–8.9)	8.6 (7.9–8.9)	8.7 (8.1–9.0)	8.7 (7.9–9.0)
N:P	31 (2–139)	32 (1–133)	24 (2–248)	30 (1–248)

Chl a, chlorophyll a concentration; TN, total nitrogen; TP, total phosphorus; PO_4_‐P, phosphate phosphorus; NO_3_‐N, nitrate nitrogen; NH_4_‐N, ammonia nitrogen; SD, Secchi depth; T, water temperature; DO, dissolved oxygen; COND, conductivity; N:P, total nitrogen to total phosphorous ratio.

### Long‐term variations in the temperature and Chl a of Lake Erhai

The air temperature around the study lake increased by approximately 1°C, and the Chl a of the lake greatly increased from 1985 to 2010 (Fig. 3A). The abundance of both phytoplankton and cyanobacteria increased greatly (phytoplankton from 64.9 to 1864.6 × 10^4^ cell·L^−1^; cyanobacteria from 35.4 to 776.8 × 10^4^ cell·L^−1^) from 1957 to 2012 (Table 3). Furthermore, since 1957, the dominance of rotifers and cladocerans increased, but that of copepods decreased (Table 3).

**Table 3:** Long‐term changes in the biotic index in Lake Erhai over the past 57 years

Years	Rotifers (ind·L^−1^)	Cladocerans (ind·L^−1^)	Copepods (ind·L^−1^)	Phytoplankton (10^4^ cell·L^−1^)	Cyanobacteria (10^4^ cell·L^−1^)	Sources
1957	54	10	120	64.9	–	Wu and Wang (1999)
1980	80	80	155	123.6	–	Wu and Wang (1999)
1987	240.7	–	–	132.9	–	Du (1989)
1992	483	17	62.3	99.6	–	Zhao et al. (2011)
1995	–	–	–	162.3	35.4	Dong (1999)
1997	52.5	5.4	8.72	563.2	–	Dong (1999)
1998	–	–	–	985	426.5	Dong (1999)
2006	–	–	–	823.3	444.3	Wang (2008)
2011–2012	153.7	44.4	38.22	1864.6	776.8	This study

### Results of the statistical analysis

**Figure 4:**
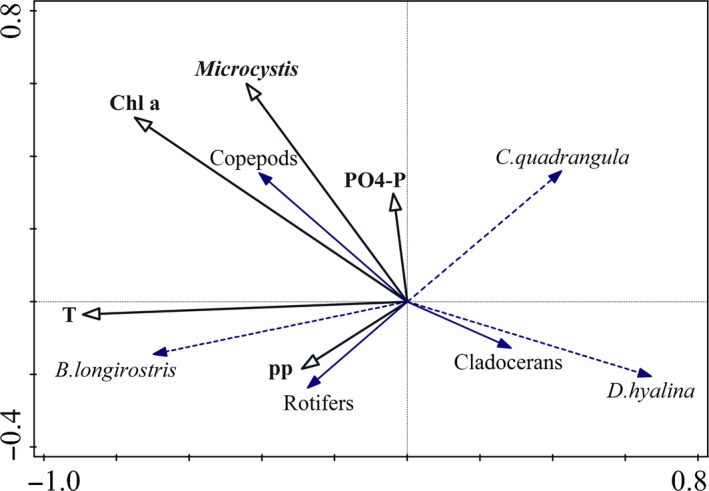
Detrended correspondence analysis ordination of significantly independent environmental variables and the zooplankton community of Lake Erhai from August 2011 to July 2012. Temporal and spatial effects on the zooplankton community data were removed using generalized linear models in which time, longitude and latitude were included as covariates. The resulting residuals were analyzed to determine the effects of specific environmental factors on the zooplankton community controlling for time and space. PO4‐P, phosphate phosphorus; pp, total phytoplankton biomass.

**Figure 5:**
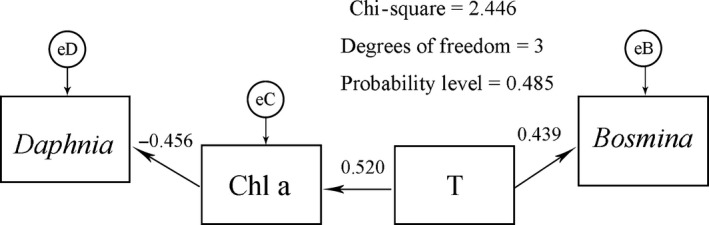
Path diagram obtained from the path analysis. Temporal and spatial effects on the zooplankton community data were removed using generalized linear models in which time, longitude and latitude were included as covariates. The resulting residuals were analyzed to determine the effects of specific environmental factors on the zooplankton community controlling for time and space. The path coefficient (number above each arrow) indicates the strength of each causal relationship; eD, eB, and eC are residual errors. The significant environmental variables were incorporated into the final model, and the details of model selection are presented in the supporting information (Appendix S1).

**Figure 6:**
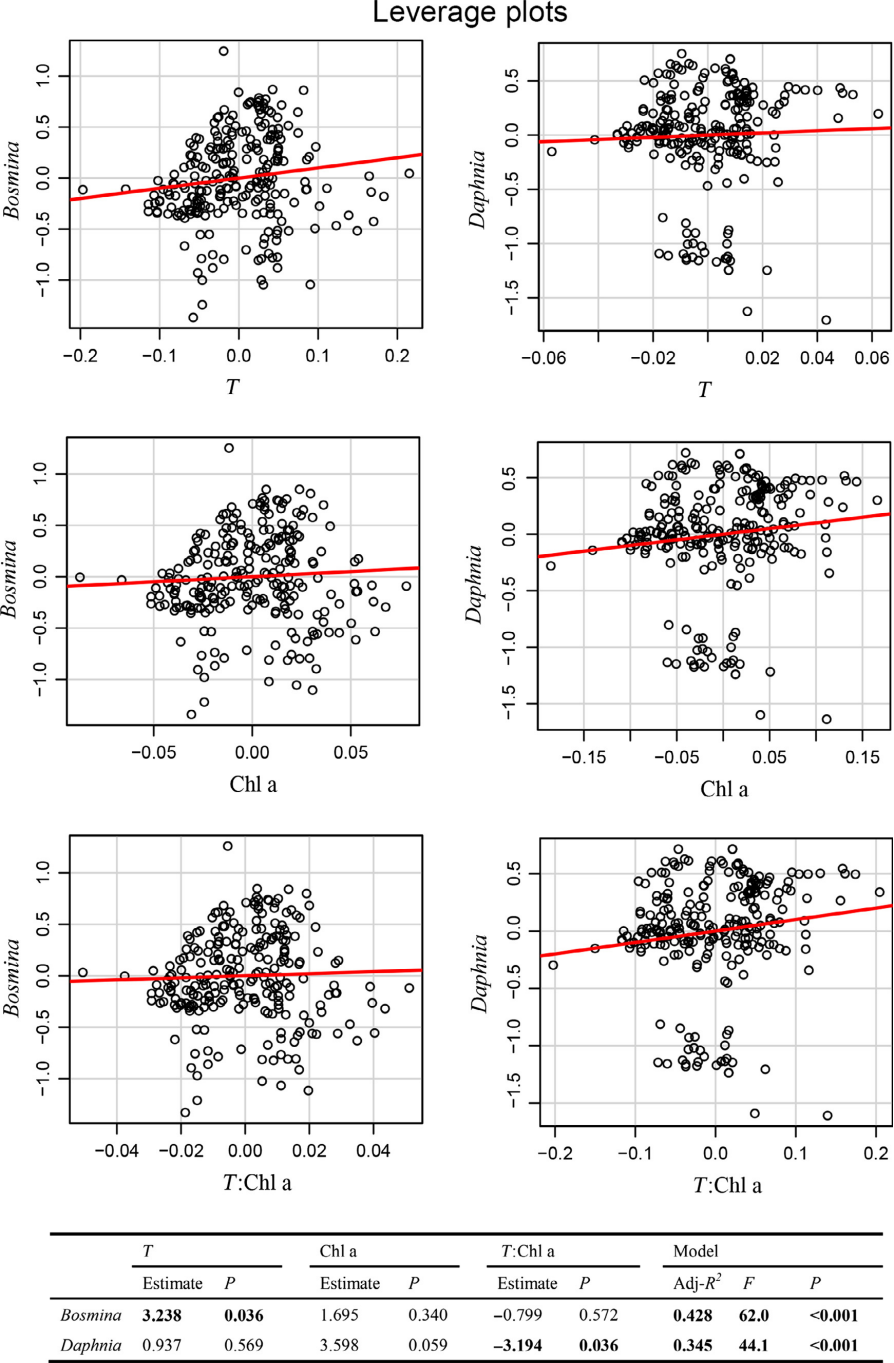
Leverage plots of the linear regressions of T and Chl a on the zooplankton community. Temporal and spatial effects on the zooplankton community data were removed using generalized linear models in which time, longitude and latitude were included as covariates. The resulting residuals were analyzed to determine the effects of T and Chl a on the zooplankton community controlling for time and space. The effect leverage plot for X is type of scatterplot of the *X*‐residuals against the *Y*‐residuals. *Y*‐axis, the residuals of *Bosmina* and *Daphnia* regressed on all of the predictors except *X*;* X*‐axis, the residuals of T, Chl a and T:Chl a regressed on all of the other predictors in the model. T:Chla, the interaction of temperature and Chl a.

Both the DCA and path analysis results showed that temperature and Chl a were the most important factors affecting the zooplankton community. The DCA indicated that temperature and Chl a were negatively related to *D. hyalina* and *C. quadrangula* but positively related to *B. longirostris* (Fig. 4). The path diagram showed that *Daphnia* was negatively affected directly by Chl a (path coefficient of −0.456) and indirectly by temperature (path coefficient of −0.237), whereas *Bosmina* was directly positively affected by temperature (path coefficient of 0.439) but relatively unaffected by Chl a (Fig. 5, Appendix S1). Furthermore, Chl a was directly positively affected by temperature (path coefficient of 0.520). The linear regressions showed that temperature was directly positively related to *Bosmina* and that the interaction of temperature and Chl a was negatively related to *Daphnia* (Fig. 6).

## Discussion

### Temperature has a dominant effect on the seasonal succession of the zooplankton community

Our results revealed that the effects of temperature on cladocerans varied depending on the species; temperature had negative impacts on *D. hyalina* and *C. quadrangula* but positive effects on *B. longirostris*. Temperature affects zooplankton life cycles, which leads to zooplankton community succession, and higher temperatures benefit *Bosmina* through increased reproductive success more than *Daphnia* (Hanazato and Masayuki 1885). Higher temperatures can theoretically promote comparable benefits for small zooplankton species because of their r strategy‐related growth pattern (Bunioto and Arcifa 2007; Xiang et al. 2010), and the relative increase in small cladocerans was most likely due to their higher tolerance of temperature fluctuations and higher optimum growth temperatures (Mason and Abdulhussein 1991; Moore et al. 1996; Kappes and Sinsch 2005). A previous study showed that the temperature dependence of the growth rate would selectively favor small species over large ones (Huntley and Lopez 1992), which further supports our results.

In conclusion, temperature had clear effects on seasonal zooplankton succession, mainly the succession of cladocerans, which shifted from *Daphnia* to *Bosmina* during the warm seasons. The effects of temperature on zooplankton might also synergistically interact with other environmental factors, such as eutrophication.

### Eutrophication strengthens zooplankton community succession

In eutrophic lakes, Chl a tends to increase with increasing nutrient availability within a certain range (Hecky and Kilham 1988; Pauly and Christensen 1995). Therefore, we investigated the effects of eutrophication using increasing Chl a levels as a proxy for eutrophication in the lake (Ptacnik et al. 2008; Jochimsen et al. 2012; Huo et al. 2013). The increase in Chl a during warm seasons typically means more food for the zooplankton, but our results showed that although the concentration of Chl a reached high levels during warm months, the biomass of *Daphnia* spp. decreased. In addition, *Daphnia* biomass decreased in the southern region although Chl a was the highest in this area. Chl a can favor *Daphnia* spp. through increased primary productivity, but our findings indicate that it can also harm *Daphnia* due to the negative effects caused by the increase in cyanobacterial blooms during warm seasons.

Succession within zooplankton communities can be expected to be triggered by increased temperatures and strengthened by the effects of increased eutrophication as a result of increases in harmful cyanobacteria. Consistent with the results of Ghadouani et al. (2003), we found that the increased abundance of *Microcystis* in summer might have disturbed the zooplankton, and our results also showed that the change in *Microcystis* populations with the increase in temperature was not gradual but increased sharply at temperatures higher than 20°C. Simultaneously, the biomass of zooplankton, especially *Daphnia*, declined during warm months. In addition, the overall zooplankton biomass and the biomass of *Daphnia* relative to *Bosmina* were both lowest in the southern region, where *Microcystis* blooms occurred in the littoral zones from August to September 2011. Sun et al. (2012) also showed that *Microcystis* promoted a shift in zooplankton composition, resulting in a higher fraction of small cladocerans. Furthermore, the low N:P and SD in the southern region might have benefitted the *Microcystis* population. Harmful cyanobacteria become dominant when N:P is less than 29:1 (Smith 1983), and N:P values are low in most subtropical lakes, which favors *Microcystis* and further enhances their negative effects on the zooplankton community (Sun et al. 2012; Zhang et al. 2013). In addition, the reduced light penetration caused by increases in phytoplankton and cyanobacterial blooms during the warm seasons will benefit *Microcystis* because of their low light resistance (Huisman et al. 2004), further affecting zooplankton succession.

Warming affects food web interactions (Rall et al. 2010), and when combined with eutrophication, promotes blooms of harmful cyanobacteria (Johnk et al. 2008). Over the long term, the structure of the zooplankton of Lake Erhai has changed since 1957, which might be due to both climate change and eutrophication. Our results showed a clear upward trend in both the air temperature around the study lake and the Chl a concentration in the lake from 1985 to 2010, and the abundance of phytoplankton and cyanobacteria both increased greatly from 1957 to 2012. A previous study showed a reduction in the proportion of *Daphnia* spp. and a comparative increase in small species in Lake Erhai over the long term (Wu and Wang 1999). The increase in cyanobacteria likely promoted the succession of the zooplankton community, which might have been accelerated markedly because of climate change and the increased eutrophication in recent decades (Thomas et al. 2004; Huang et al. 2013; Liu et al. 2014).

## Conclusion

In summary, elevated temperatures will alter the composition of zooplankton species, primarily the cladocerans, decrease the zooplankton biomass, and shift the dominant genus from *Daphnia* to *Bosmina* during the warm seasons; such changes are expected to be strengthened by eutrophication. The effects of temperature variations and eutrophication on zooplankton communities have likely occurred in conjunction with harmful cyanobacterial blooms. Moreover, long‐term climate change and increased eutrophication can promote the dominance of small species, and this succession might be markedly accelerated under climate change and the increased eutrophication observed in recent decades.

## Conflict of Interest

None declared.

## Supporting information


**Appendix S1.** Details of model selection of path analysis.
