# Supplementary material for: Eutrophication strengthens the response of zooplankton to temperature changes in a high‐altitude lake
Source: Ecol Evol. 2016 Aug 30;6(18):6690–701. doi: 10.1002/ece3.2308 (PMC5058538; doi:10.1002/ece3.2308)
Supplement: Supplementary file 1 — Appendix S1. Details of model selection of path analysis. [file ECE3-6-6690-s001.docx]

**Appendix S1.** Details of model selection of path analysis using maximum likelihood estimates. Significant effects are indicated in bold. Multiple hypotheses were conducted to select the significant variables in the final model.

**1 Select important causal factos for Daphnia and Bosmina respectively**

**Regression Weights:**

|  |  |  | Estimate | S.E. | C.R. | P |
| --- | --- | --- | --- | --- | --- | --- |
| Daphnia | <--- | NH4 | -.798 | .389 | -2.054 | .040 |
| Daphnia | <--- | WD | .151 | .080 | 1.885 | .059 |
| **Daphnia** | **<---** | **Chla** | **-.322** | **.048** | **-6.732** | ******* |
| Daphnia | <--- | T | .016 | .095 | .170 | .865 |
| Daphnia | <--- | NP | -.023 | .037 | -.624 | .533 |
| Daphnia | <--- | TP | -.936 | .652 | -1.436 | .151 |
| Daphnia | <--- | TN | -.027 | .172 | -.157 | .875 |
| Daphnia | <--- | PO4 | .929 | 2.135 | .435 | .664 |
| Daphnia | <--- | SD | .233 | .105 | 2.223 | .026 |
| Daphnia | <--- | NO3 | .041 | .221 | .185 | .853 |

|  |  |  | Estimate | S.E. | C.R. | P |
| --- | --- | --- | --- | --- | --- | --- |
| Bosmina | <--- | NO3 | -.026 | .086 | -.301 | .764 |
| Bosmina | <--- | Chla | .016 | .019 | .870 | .384 |
| Bosmina | <--- | NH4 | -.357 | .152 | -2.351 | .019 |
| Bosmina | <--- | NP | -.032 | .014 | -2.224 | .026 |
| Bosmina | <--- | TN | .078 | .067 | 1.157 | .247 |
| Bosmina | <--- | SD | -.029 | .041 | -.702 | .483 |
| Bosmina | <--- | WD | -.010 | .031 | -.324 | .746 |
| **Bosmina** | **<---** | **T** | **.281** | **.037** | **7.571** | ******* |
| Bosmina | <--- | PO4 | 1.450 | .835 | 1.736 | .083 |
| Bosmina | <--- | TP | -.580 | .255 | -2.275 | .023 |

**2 Select important causal factos for Chla**

**Regression Weights:**

|  |  |  | Estimate | S.E. | C.R. | P |
| --- | --- | --- | --- | --- | --- | --- |
| Chla | <--- | TN | .148 | .191 | .777 | .437 |
| Chla | <--- | TP | .497 | .725 | .686 | .493 |
| Chla | <--- | NO3 | .466 | .246 | 1.894 | .058 |
| Chla | <--- | NH4 | .258 | .432 | .598 | .550 |
| Chla | <--- | PO4 | 4.923 | 2.373 | 2.074 | .038 |
| Chla | <--- | NP | -.019 | .041 | -.464 | .643 |
| **Chla** | **<---** | **T** | **1.019** | **.106** | **9.657** | ******* |

**3 The final model of path analysis**

**

**

##### Result (Default model)

##### Chi-square = 2.446

##### Degrees of freedom = 3

##### Probability level = .485

##### Standardized Total Effects (Group number 1 - Default model)

|  | T | Chla |
| --- | --- | --- |
| Chla | .520 | .000 |
| Bosmina | .439 | .000 |
| Daphnia | -.237 | -.456 |

##### Standardized Direct Effects (Group number 1 - Default model)

|  | T | Chla |
| --- | --- | --- |
| Chla | .520 | .000 |
| Bosmina | .439 | .000 |
| Daphnia | .000 | -.456 |

##### Standardized Indirect Effects (Group number 1 - Default model)

|  | T | Chla |
| --- | --- | --- |
| Chla | .000 | .000 |
| Bosmina | .000 | .000 |
| Daphnia | -.237 | .000 |
